# Risk factors of metachronous recurrence after endoscopic submucosal dissection for superficial esophageal squamous cell carcinoma

**DOI:** 10.1371/journal.pone.0238113

**Published:** 2020-09-04

**Authors:** Ga Hee Kim, Yang Won Min, Hyuk Lee, Byung-Hoon Min, Jun Haeng Lee, Poong-Lyul Rhee, Jae J. Kim

**Affiliations:** 1 Department of Medicine, Samsung Medical Center, Sungkyunkwan University School of Medicine, Seoul, Republic of Korea; 2 Department of Gastroenterology, Samsung Medical Center, Sungkyunkwan University School of Medicine, Seoul, Republic of Korea; University Hospital Hamburg Eppendorf, GERMANY

## Abstract

Esophageal endoscopic submucosal dissection (ESD) can be a curative treatment for superficial esophageal squamous cell carcinoma (SESCC). However, it is unclear whether the development of metachronous recurrence after ESD may be explained based on several risk factors. This study aimed to assess the incidence and the risk factors of metachronous recurrence of SESCC after ESD. This retrospective analysis was conducted at Samsung Medical Center, Seoul, Korea, from April 2007 to May 2018. Two hundred and fifty-three SESCC patients treated with ESD were followed using surveillance endoscopy after the procedure. Risk factors for metachronous esophageal SCC were analyzed using the Kaplan-Meier method and Cox’s proportional hazards model. Metachronous esophageal SCCs were found in 21 (8.3%) of the 253 patients. Six patients (2.4%) with extraesophageal recurrence such as lymph node metastasis confirmed by imaging were excluded from patients with metachronous recurrence and data were censored from the recurrence date. Univariate analysis revealed that the presence of many (>10) irregularly shaped multiform Lugol-voiding lesions (LVLs) around the main lesion, margin of the main LVL, and tumor differentiation were risk factors for the development of metachronous cancer. Multivariate analysis also revealed that many (>10) LVLs (hazard ratio [HR], 6.32; 95% confidence interval [CI], 1.62–24.72; *p* = 0.047) and unclear or spiculated margin of the main LVL (HR, 6.51; 95% CI, 1.44–29.42; *p* = 0.029) were associated with the risk of metachronous recurrence. Metachronous esophageal SCC develops in patients treated with ESD for SESCC. A risk assessment is important for surveillance before and after ESD for SESCC. Number of LVLs and tumor edge type are associated with an increased risk of metachronous cancer in SESCC. Patients will benefit from careful endoscopic surveillance when endoscopists pay attention to these tumor characteristics.

## Introduction

Esophageal cancer is the sixth most common cause of cancer-related deaths worldwide [1]. Squamous cell carcinoma (SCC) is the major type of esophageal cancer in parts of Asia. Early stage superficial esophageal squamous cell carcinoma (SESCC) is more frequently detected with the development of techniques for endoscopic diagnosis [2, 3].

Endoscopic resection is a potentially curative treatment with minimal invasiveness for SESCC, and many studies demonstrated its favorable long-term outcomes [4]. However, it leaves a larger area of esophageal mucosa than does surgery, and metachronous SCC can occur in the preserved esophageal mucosa after endoscopic resection for SESCC [5]. To determine the possibility of a recurrence of metachronous SCCs after initial endoscopic resection, careful surveillance is needed.

A few Japanese studies investigated the incidence of and risk factors for metachronous SCC following endoscopic treatment for SESCC. The reported incidence is 12–35% [5, 6]. Male sex, alcohol consumption, smoking, multiple Lugol-voiding lesions (LVLs), and single nucleotide polymorphisms in aldehyde dehydrogenase-2 and alcohol dehydrogenase 1B were associated with metachronous recurrence [7, 8]. However, the involved sample sizes were rather small and the associations between endoscopic findings and recurrence risk have not been well investigated. Thus, this study aimed to assess the incidence of metachronous recurrence following endoscopic submucosal dissection (ESD) for SESCC and identify novel related endoscopic features using a large cohort.

## Methods

### Patients

**Fig 1:**
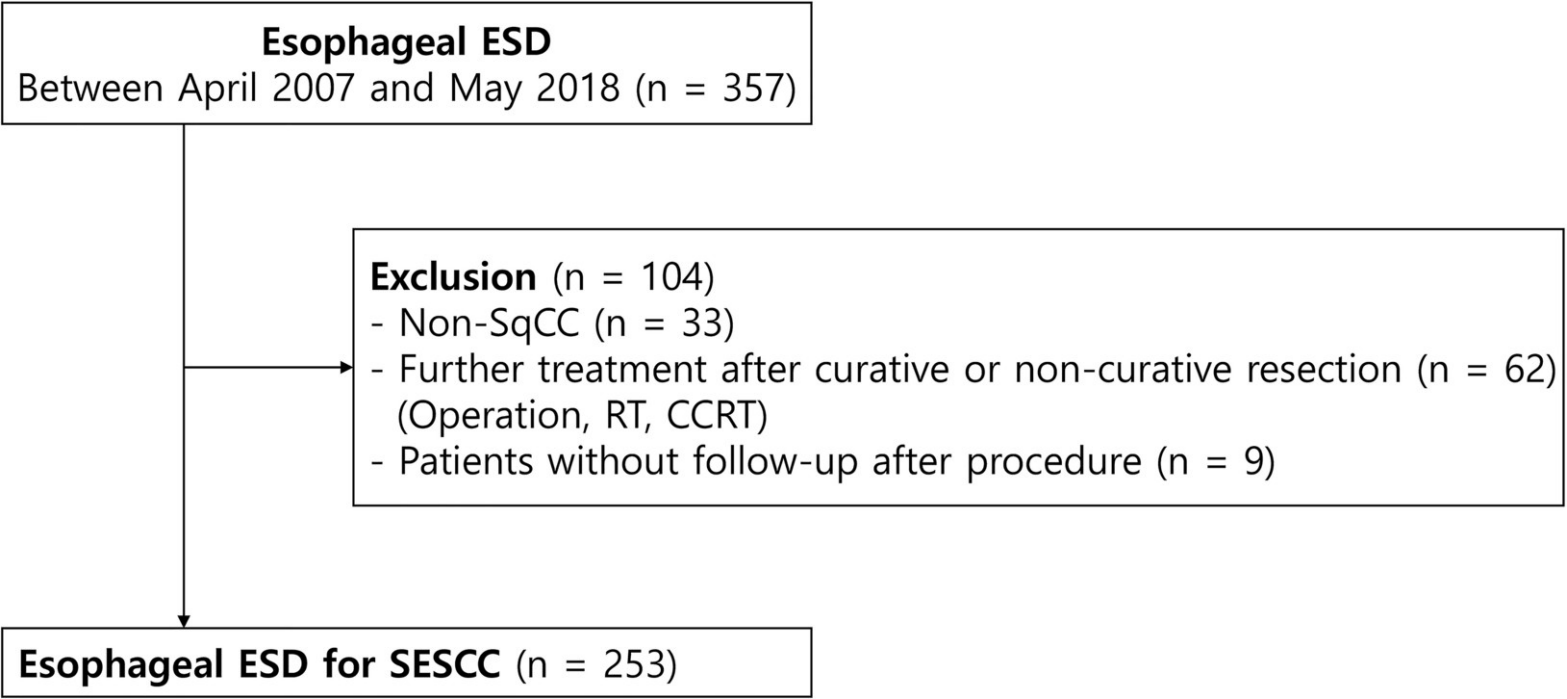
Flow diagram of the study. ESD, endoscopic submucosal dissection; RT, radiotherapy; CCRT, concurrent chemoradiotherapy; SESCC, superficial esophageal squamous cell carcinoma; SCC, squamous cell carcinoma

Three hundred fifty-seven consecutive patients who underwent ESD for esophageal cancer at Samsung Medical Center, Seoul, Korea, were enrolled in this retrospective cohort study from April 2007 to May 2018. We excluded 33 patients diagnosed with non-SCC based on ESD specimens, 62 patients with further treatment after endoscopic resection (esophageal surgery, radiation therapy, chemoradiotherapy), and 9 patients without follow-up. Therefore, a total of 253 patients with ESCC were enrolled (Fig 1). We defined curative resection as when cancer was confined to the mucosal layer, had no lymphovascular invasion, and no resection margin positivity. Among the 253 paients of the study, 41 had noncurative resection due to lymphovascular invasion (n = 11), SM invasion (n = 40), and both (n = 10). 212 (84%) achieved curative resection, however, en-bloc and complete resection was achieved in all patients (n = 253).

### Endoscopic procedure and follow-up

Patients enrolled in this study have no history of previous esophageal ESD. Prior to the 1st ESD, all patients underwent an endoscopic evaluation including chromoendoscopy with narrow-band imaging (NBI) and the Lugol’s dye spray method. NBI can avoid discomfort in patients, such as pain caused by esophageal mucosal damage or severe allergic reactions that may occur after iodine staining. In addition, it is possible to observe the detailed vascular structure of the tumor surface with magnifying endoscopy with NBI, and through this, the intraepithelial papillary capillary loop pattern classification can be used to predict the invasion depth of superficial esophageal cancer [9]. In Lugol’s dye spray method, approximately 10mL of a 1% Lugol iodine solution was sprayed over the entire esophageal mucosa with a catheter after a conventional endoscopic examination. As this iodine-based absorptive staining has an affinity for glycogen in non-keratinized squamous epithelium, it is useful for the identification of squamous neoplasms [10]. Iodine staining is a useful method for diagnosing early esophageal cancer in the high-risk group with esophageal cancer and microscopic mucosal changes. It also has the advantage of being able to determine the exact border of the lesion, which is a great help in determining the extent of resection. In addition, a computed tomography (CT) scan of the chest and/or positron emission tomography-CT (PET-CT) were performed to identify possible distant or lymph node metastases. Esophageal ESD was performed using a standard technique as described elsewhere [11, 12]. In the beginning, the endoscopist marked 2–3 mm away from the edge of the cancer, which is well determined by Lugol’s iodine chromoendoscopy. After the submucosal injection, circumferential mucosal pre-cutting was performed. The submucosal layer under the lesion was dissected using various types of ESD knives after elevation of the lesion by the submucosal injection.

For follow-up, upper endoscopy was performed at 2 months after ESD to exclude the presence of any residual tumor. Endoscopy and chest and abdomen CT scans were then performed every 6 months for the first 3 years and then annually until the fifth year after ESD.

### Data collection and definitions

We used a prospectively collected database of patients who underwent esophageal ESD. These data included demographic parameters (e.g. patient age, sex, body mass index, smoking and alcohol status, and past medical history), tumor characteristics (e.g. endoscopic tumor morphology; LVLs; margin of main LVL; tumor location, circumference, size, and pathology; differentiation, depth and lymphovascular invasion). The study was approved and the need for informed consent was waived by the institutional review board of Samsung Medical Center (IRB #: SMC 2020-05-114-001).

**Fig 2:**
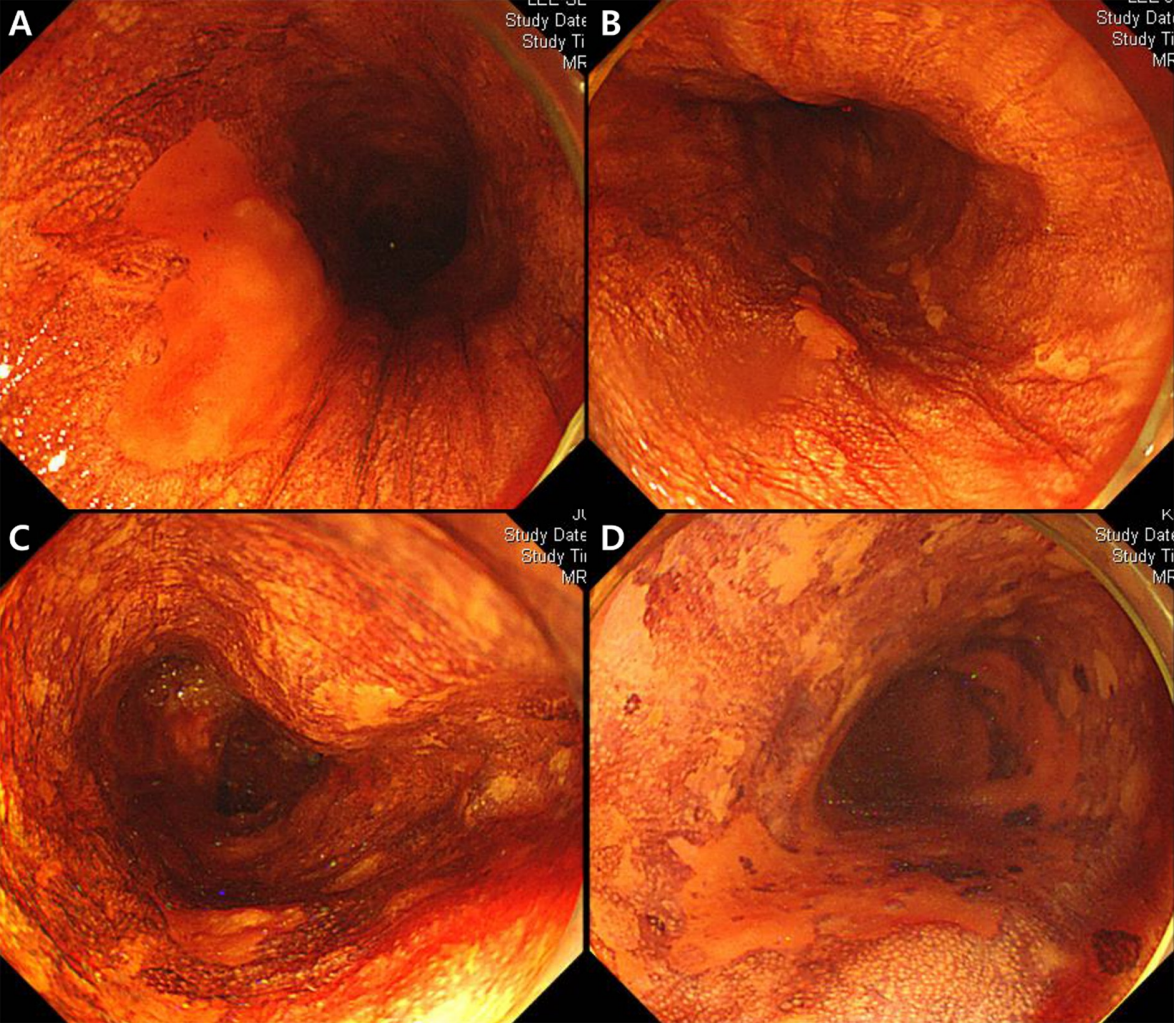
Endoscopic images of Lugol chromoendoscopy. (A) No Lugol voiding lesions (LVLs); (B) several (≤10) small LVLs; (C) many (>10) LVLs; (D) many (>10) irregularly shaped multiform LVLs around the main lesion.

**Fig 3:**
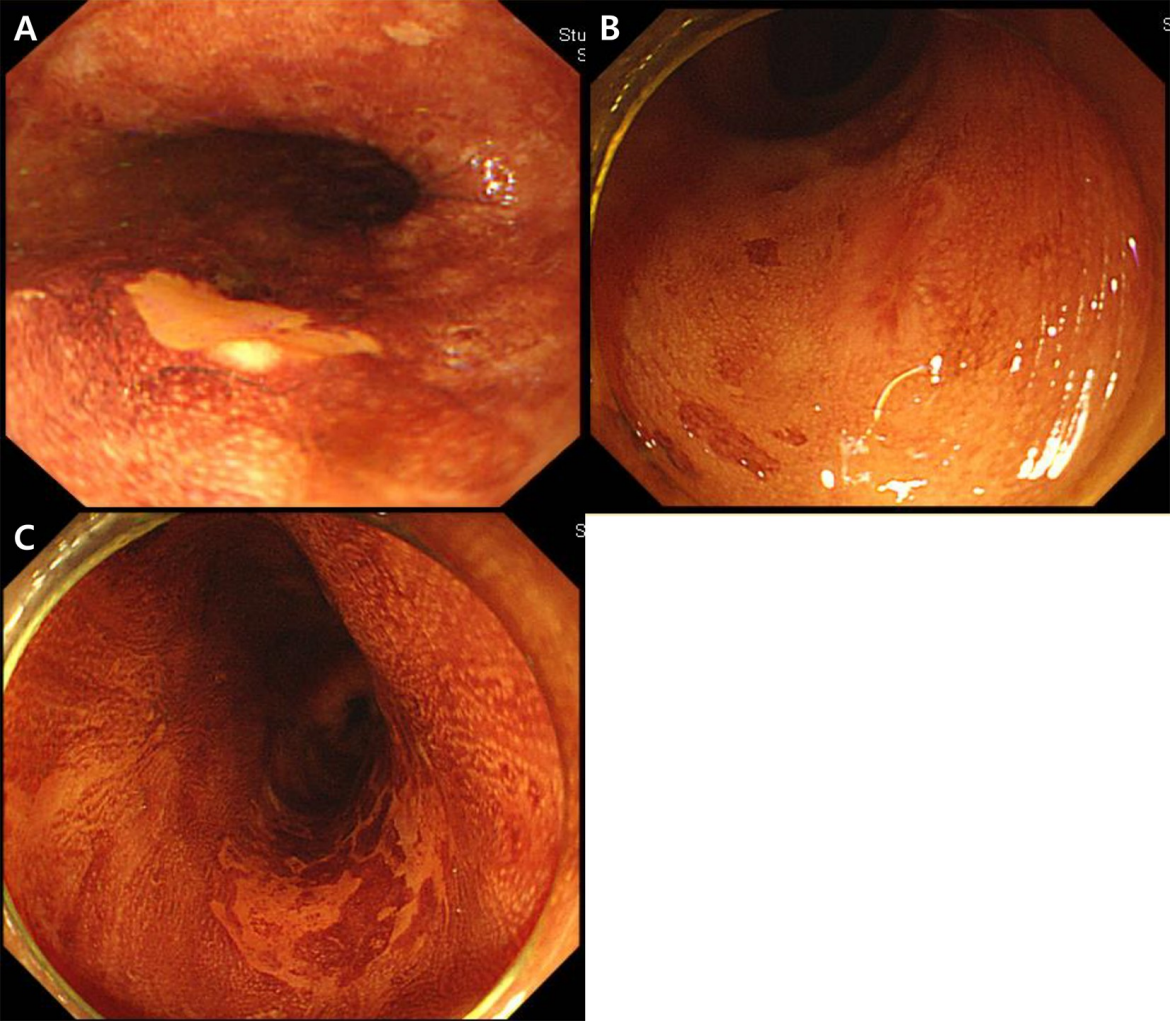
Margin of main Lugol-voiding lesion (LVL). (A) clear margin of the main LVL; (B) unclear margin of the main LVL; (C) spiculated margin of the main LVL.

Metachronous recurrence was defined as histologically proven SESCC at another site of the ESD scar after ESD. Patients were divided into 4 groups based on the number and multiform patterns of LVLs in the background esophageal mucosa as follows: A, no LVL; B, several (≤10) small LVLs; C, many (>10) LVLs; D, multiple (>10) irregularly shaped LVLs [13, 14]. Based on the margin of the main LVL, patients were also divided into 3 groups as follows: a, clear; b, unclear; and c, spiculated (Figs 2 and 3).

### Statistical analysis

Data are expressed as mean ± standard deviation (SD) or number (%). We performed univariate analysis using the Wald chi-squared test, Cox’s proportional hazards model, and the Kaplan-Meier method for metachronous recurrence. Multivariate analysis was performed with selection of variables with at least *p* < 0.2 on univariate analysis. *p* values less than 5% were considered statistically significant. All statistical analyses were executed using SAS version 9.4 (SAS Institute, Cary, NC, USA).

## Results

### Clinicopathologic characteristics

The clinicopathological characteristics of the patients with versus without metachronous recurrence of SESCC after ESD are shown in Table 1. A total of 21 (8.3%) patients experienced metachronous recurrence. Among these patients, the mean age was 66.2 ± 8.2 years; 19 (90.5%) patients were men. Endoscopic findings of 4 (19.0%), 9 (42.9%), 3 (14.3%), and 5 (23.8%) patients with metachronous recurrence represented no LVLs, several (≤10) small LVLs, many (>10) LVLs, and many (>10) irregularly shaped multiform LVLs around the main lesion, respectively. The mean tumor size of the resected specimen was 1.5 ± 0.8 cm. Most tumors (61.9%) had moderately differentiated tumors and negative lymphovascular invasion (90.5%). Regarding the margin of the main LVL, 2 (9.5%), 5 (23.7%), and 14 (66.7%) lesions were classified as clear, unclear, and spiculated, respectively.

**Table 1:** Baseline characteristics of patients with versus without metachronous recurrence.

Characteristics	Metachronous recurrence		*p-*value
	No (n = 232)	Yes (n = 21)	
**Age (years)**	65.0 ± 8.0	66.2 ± 8.2	0.28
**Sex (male)**	219 (94.4)	19 (90.5)	0.66
**BMI (kg/*m***^**2**^**)**	23.6 ± 2.9	23.5 ± 3.9	0.69
**Smoking**			0.89
Current smoker	36 (15.5)	4 (19.0)	
Ex-smoker	136 (58.6)	10 (47.6)	
Never-smoker	60 (25.9)	7 (33.3)	
**Quit smoking after procedure**	161 (69.4)	13 (61.9)	0.87
**Heavy alcohol intake (>4 days/week)**	29 (12.5)	4 (19.0)	0.27
**Alcohol intake after procedure**			0.67
None	165 (71.1)	16 (76.2)	
Decreased before procedure	34 (14.7)	2 (9.5)	
Did not decrease before procedure	33 (14.2)	3 (14.3)	
**DM**	45 (19.4)	3 (14.3)	0.81
**HTN**	81 (34.9)	5 (23.8)	0.72
**Lugol-voiding lesion** **(around the main lesion)**			0.04
No LVLs	92 (39.7)	4 (19.0)	
Several (≤10) small LVLs	82 (35.3)	9 (42.9)	
Many (>10) LVLs	38 (16.4)	3 (14.3)	
Many (>10) irregularly-shaped multiform LVLs	20 (8.6)	5 (23.8)	
**Tumor location**			0.95
Upper thoracic	24 (10.3)	1 (4.8)	
Middle thoracic	62 (26.7)	7 (33.3)	
Lower thoracic	143 (61.6)	13 (61.9)	
EG junction	3 (1.3)	0 (0.0)	
**Tumor size**	1.7 ± 1.1	1.5 ± 0.8	0.86
**Tumor depth**			0.77
Intraepithelial, M1	40 (17.2)	4 (19.0)	
LP, M2	97 (41.8)	7 (33.3)	
MM, M3	58 (25.0)	7 (33.3)	
SM1 (<200 μm)	5 (2.2)	0 (0.0)	
SM2, 3	32 (13.8)	3 (14.3)	
**Tumor differentiation**			<0.0001
Well	47 (20.3)	6 (28.6)	
Moderate	185 (79.7)	13 (61.9)	
Poor	0 (0.0)	2 (9.5)	
**Lymphovascular invasion**	9 (3.9)	2 (9.5)	0.16
**Margin of main Lugol-voiding lesion**			0.04
Clear	100 (43.1)	2 (9.5)	
Unclear	32 (13.79)	5 (23.8)	
Spiculated	100 (43.1)	14 (66.7)	
**Tumor circumference**			0.67
<1/4	67 (28.9)	5 (23.8)	
1/4-2/4	137 (59.1)	16 (79.2)	
2/4-3/4	24 (10.3)	0 (0.0)	
≥3/4	4 (1.7)	0 (0.0)	

### Univariate and multivariate analysis of metachronous recurrence

**Fig 4:**
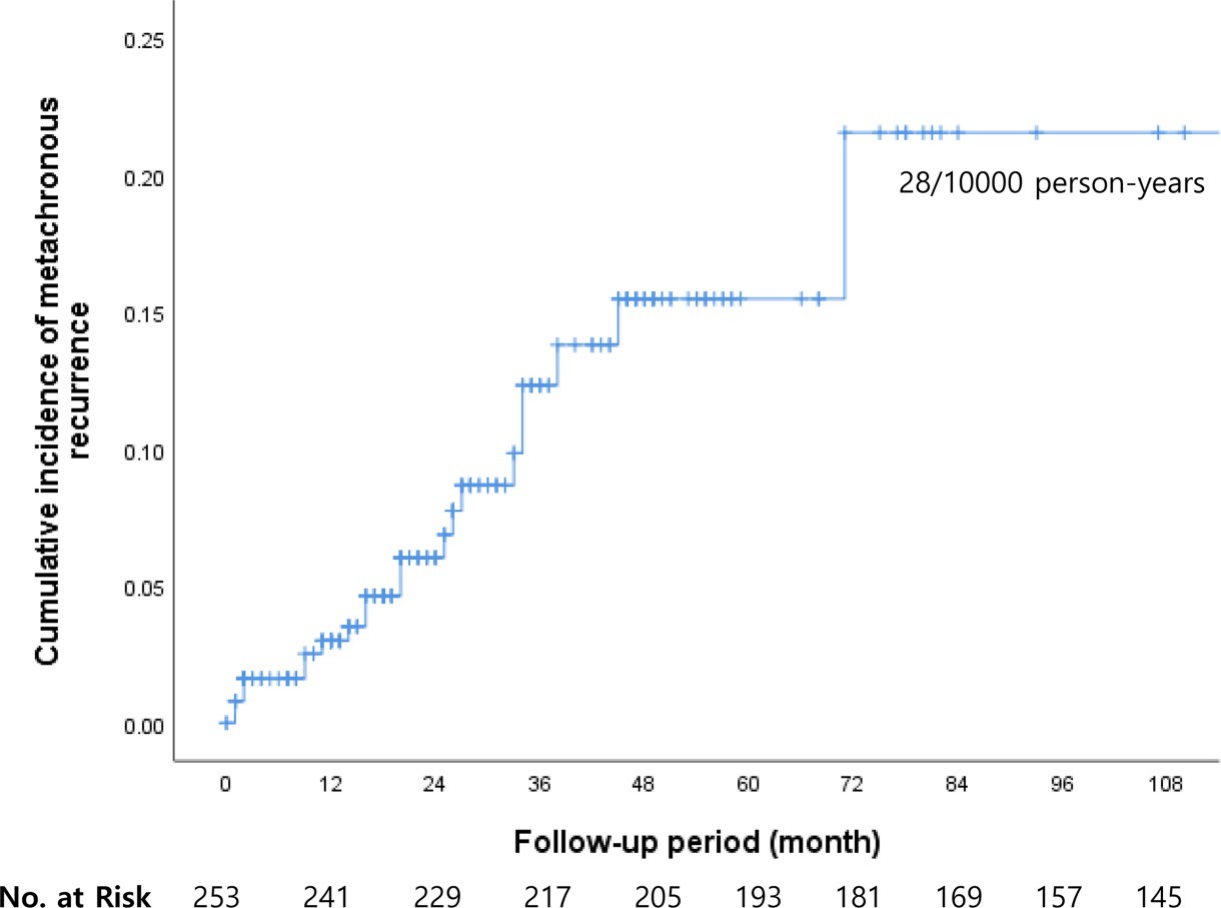
Metachronous recurrence after endoscopic submucosal dissection for superficial esophageal squamous cell carcinoma occurred in 8.3% of the patients allocated to surveillance (incidence, 28/10000 person-years).

The cumulative incidence of metachronous recurrence was 8.3% of the patients allocated for surveillance. The incidence was 28/10000 person-years (Fig 4).

The univariate analysis of the risk factors of metachronous recurrence after endoscopic resection is shown in Table 2. The metachronous recurrence rate was higher in patients with many (>10) irregularly shaped multiform LVLs around the main lesion (*p* = 0.04), poor tumor differentiation (*p* < 0.0001), and a spiculated margin of the main LVL (*p* = 0.04).

**Table 2:** Univariate analysis of the risk factors for metachronous recurrence after endoscopic resection.

Variable	Univariate analysis	
	Hazard Ratio (95% CI)	*p* value
**Age (years)**	1.03 (0.98–1.09)	0.28
**Male sex**	1.39 (0.32–6.02)	0.66
**BMI (kg/*m***^**2**^**)**	0.97 (0.83–1.13)	0.69
**Smoking**		0.89
Current smoker	1	
Ex-smoker	0.80 (0.25–2.60)	
Never smoker	1.00 (0.29–3.44)	
**Quit smoking after procedure**	0.84 (0.11–6.46)	0.87
**Heavy alcohol intake (>4 days/week)**	1.75 (0.64–4.79)	0.27
**Alcohol intake after procedure**		0.67
None	1	
Decreased before the procedure	0.53 (0.12–2.30)	
Did not decrease before procedure	0.79 (0.23–2.72)	
**DM**	0.86 (0.25–2.94)	0.81
**HTN**	0.83 (0.30–2.30)	0.72
**Lugol-voiding lesion (around the main lesion)**		0.04
No LVLs	1	
Several (≤10) small LVLs	3.46 (1.06–11.29)	
Many (>10) LVLs	3.19 (0.70–14.44)	
Many (>10) irregular-shaped multiform LVLs	6.76 (1.80–25.48)	
**Tumor location**		0.95
Upper thoracic	1	
Middle thoracic	1.78 (0.28–11.19)	
Lower thoracic	1.49 (0.25–8.72)	
EG junction	2.20 (0.08–64.25)	
**Tumor size**	0.96 (0.59–1.56)	0.86
**Tumor depth**		0.77
Intraepithelial, M1	1	
LP, M2	0.72 (0.21–2.50)	
MM, M3	1.25 (0.36–4.31)	
SM1 (<200 μm)	1.04 (0.05–23.27)	
SM2, 3	1.69 (0.37–7.71)	
**Tumor differentiation**		<0.0001
Well	1	
Moderate	0.60 (0.23–1.60)	
Poor	19.19 (3.61–102.01)	
**Lymphovascular invasion**	2.85 (0.66–12.31)	0.16
**Margin of main Lugol-voiding lesion**		0.04
Clear	1	
Unclear	6.73 (1.31–34.71)	
Spiculated	6.62 (1.50–29.17)	
**Tumor circumference**		0.67
<1/4	1	
1/4-2/4	1.30 (0.47–3.58)	
2/4-3/4	0.37 (0.02–7.90)	
≥3/4	4.24 (0.18–100.52)	

**Fig 5:**
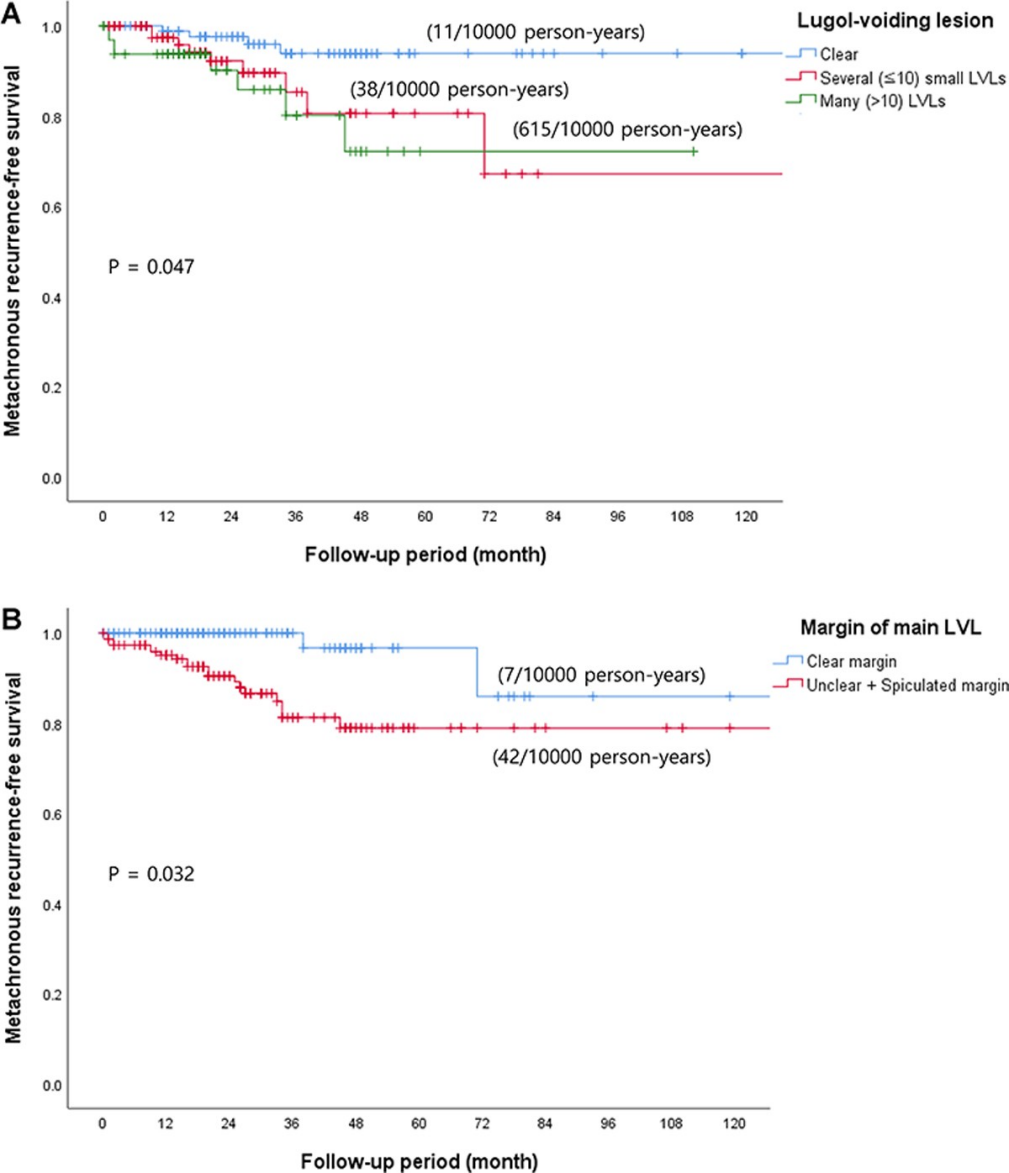
Metachronous recurrence-free survival in patients who underwent endoscopic resection for (A) Lugol-voiding lesion (LVL) and (B) Margin of the main LVL.

The multivariate analysis was performed for variables with values of *P* less than 0.2, resulting in an increased metachronous recurrence rate for patients with many (>10) LVLs and an unclear or spiculated margin of the main LVL (hazard ratio [HR], 6.32; 95% confidence interval [CI], 1.62–24.72; *p* = 0.047 and HR, 6.51; 95% CI, 1.44–29.42; *p* = 0.032, respectively; Table 3 and Fig 5).

**Table 3:** Multivariable analysis of risk factors for metachronous recurrence after endoscopic resection.

Variable	Multivariate analysis	
	Hazard ratio (95% CI)	*p* value
**Lugol-voiding lesion (around the main lesion)**		0.047
No LVLs	1	
Several (≤10) small LVLs	4.39(1.31–14.75)	
Many (>10) LVLs	6.32(1.62–24.72)	
**Margin of main Lugol-voiding lesion**		0.032
Clear	1	
Unclear + spiculated	6.51(1.44–29.42)	
**Lymphovascular invasion**	4.33(0.94–19.89)	0.059

## Discussion

Esophageal ESD has become an accepted minimally invasive treatment for SESCC. However, it leaves a larger area of esophageal mucosa than does surgery, and metachronous SCCs can occur in the preserved esophageal mucosa after endoscopic resection for SESCC [5]. Metachronous recurrence of SESCC after esophageal ESD occurred in 8.3% of the patients in our study versus 2–14% of patients in previous studies [5, 15]. There are several independent risk factors associated with recurrence, including male sex, low BMI, alcohol consumption, smoking, multiple LVLs, single nucleotide polymorphisms in aldehyde dehydrogenase-2 and alcohol dehydrogenase 1B, and treatment history of (sub)circumferential ESD [7, 8, 16]. This study also showed that morphological features of tumors such as number of LVLs and tumor edge type are associated with an increased risk of metachronous cancer in SESCC. Therefore, to determine the possibility of metachronous SCC recurrence after the initial endoscopic resection, careful surveillance is needed, especially in patients with the above mentioned risk factors.

The endoscopic findings by Lugol chromoendoscopy are useful for the detection of early-stage esophageal cancer and the risk estimation of metachronous recurrence. Previous studies demonstrated that the presence of many irregularly shaped multiform LVLs is associated with both synchronous and metachronous SESCC in patients with head and neck squamous cell carcinoma [17]. In this background, Lugol chromoendoscopy was performed before esophageal ESD to measure the procedure extent and determine the lesion appearance. In this study, we reviewed and confirmed the tumor edge type and number of LVLs around the main lesion using Lugol chromoendoscopy. Our findings demonstrated that the risk can be predicted not only by the number of LVLs but also by tumor edge type, so it is necessary to further distinguish the morphological features of the main lesion.

Shimizu et al. [5] reported that the metachronous recurrence rate of esophageal cancer was higher in patients in whom the Lugol voiding pattern of the background mucosa was scattered with a large number of patterns as opposed to the uniform type. The areas unstained by Lugol solution are histologically inflammatory lesions or various degrees of epithelial dysplastic lesions [18, 19]. Previous studies have suggested a correlation between dysplasia and carcinoma and that dysplasia is a precursor lesion of carcinoma [20]. This study assumed that these atypical epithelia were involved in the development of carcinoma during the course of field cancerization. Therefore, we assessed the number of LVL around the cancer as a risk factor for metachronous recurrence in the present study. As a result, we noticed that the number of LVL is an independent risk factor for recurrence in a multivariable analysis.

This study has some limitations. First, it was a single-center retrospective study. Because of its small cohort, it was difficult to perform a detailed subgroup analysis and test small differences with sufficient power. Second, smoking and alcohol habits did not correlate with the recurrence of SESCC in this study, although previous studies reported smoking, alcohol consumption, and dietary habits predicted the development of metachronous SESCC after endoscopic resection [21]. This may have been difficult to verify objectively because the patients were asked directly. Finally, in reviewing endoscopic feature such as LVLs, intra-observer variation may exist. Despite these limitations, this study distinguished the morphological features of tumors that can be identified by endoscopists.

In conclusion, risk assessment is important for the surveillance of the development of metachronous SESCC before and after endoscopic resection of SESCC. This study suggests that the number of LVLs and tumor edge type are associated with an increased risk of metachronous cancer in SESCC. Patients will benefit from careful endoscopic surveillance when endoscopists pay attention to these tumor characteristics.

## Supporting information

S1 File (XLSX)
